# Pre‐ and post‐stroke oral antithrombotics and mortality in patients with ischaemic stroke

**DOI:** 10.1002/pds.5530

**Published:** 2022-09-09

**Authors:** Luis A. García Rodríguez, David Gaist, Yanina Balabanova, Gunnar Brobert, Mike Sharma, Lucía Cea Soriano

**Affiliations:** ^1^ Pharmacoepidemiology Spanish Centre for Pharmacoepidemiologic Research (CEIFE) Madrid Spain; ^2^ Research Unit for Neurology Odense University Hospital, Denmark & University of Southern Denmark Odense Denmark; ^3^ Integrated Evidence Generation Bayer AG Berlin Germany; ^4^ Integrated Evidence Generation Bayer AB Stockholm Sweden; ^5^ Division of Neurology, Department of Medicine Population Health Research Institute McMaster University Hamilton Canada; ^6^ Department of Public Health and Maternal and Child Health, Faculty of Medicine Complutense University of Madrid Madrid Spain

**Keywords:** anticoagulants, ischemic stroke, general practice, atrial fibrillation

## Abstract

**Background:**

Reducing stroke occurrence requires the effective management of cardiovascular and other stroke risk factors.

**Purpose:**

To describe pre‐ and post‐stroke medication use, focusing on antithrombotic therapy and mortality risk, in individuals hospitalised for ischaemic stroke (IS) in the United Kingdom.

**Method:**

Using primary care electronic health records from the United Kingdom, we identified patients hospitalised for IS (July 2016–September 2019) and classed them into three groups: atrial fibrillation (AF) diagnosed pre‐stroke, AF diagnosed post‐stroke, and non‐AF stroke (no AF diagnosed pre‐/post‐stroke). We determined use of cardiovascular medications in the 90 days pre‐ and post‐stroke and calculated mortality rates.

**Results:**

There were 3201 hospitalised IS cases: 76.2% non‐AF stroke, 15.7% AF pre‐stroke, and 8.1% AF post‐stroke. Oral anticoagulant (OAC) use increased between the pre‐ and post‐stroke periods as follows: 54.3%–78.7% (AF pre‐stroke group), 2.3%–84.8% (AF post‐stroke group), and 3.4%–7.3% (non‐AF stroke group). Corresponding increases in antiplatelet use were 30.8%–35.4% (AF pre‐stroke group) 38.5%–47.5% (AF post‐stroke group), and 37.5%–87.3% (non‐AF stroke group). Among all IS cases, antihypertensive use increased from 66.8% pre‐stroke to 78.8% post‐stroke; statin use increased from 49.6%–85.2%. Mortality rates per 100 person‐years (95% CI) were 17.30 (14.70–20.35) in the AF pre‐stroke group and 9.65 (8.81–10.56) among all other stroke cases.

**Conclusion:**

Our findings identify areas for improvement in clinical practice, including optimising the level of OAC prescribing to patients with known AF, which could potentially help reduce the future burden of stroke.

**Key Points**

- Our study describes current management of cardiovascular risk factors among patients with ischaemic stroke in the United Kingdom.
- 54.5% of patients with atrial fibrillation (AF) pre‐stroke received oral anticoagulant therapy in the 90 days pre‐stroke versus 78.7% in the 90 days post‐stroke.
- Among all stroke cases, antihypertensive use increased from 66.8% pre‐stroke to 78.8% post‐stroke; statin use increased from 49.6% to 85.2%.
- Antiplatelet use among the non‐AF stroke group increased from 37.5% pre‐stroke to 87.3% post‐stroke.
- Mortality rates per 100 person‐years (95% CI) were 17.30 (14.70–20.35) in the AF pre‐stroke group and 9.65 (8.81–10.56) among all other stroke cases.

**Plain Language Summary**

The absolute number of people experiencing ischaemic stroke (IS) is increasing, and good management of cardiovascular risk factors is needed to reduce levels of IS risk in the general population. Using primary care electronic health records from the United Kingdom, we calculated mortality rates among patients with IS, and described the use of cardiovascular medications in these patients, both before and after their stroke. We identified 3201 patients who had been hospitalised for IS: 15.7% had a diagnosis of atrial fibrillation (AF) pre‐stroke, 8.1% had AF post‐stroke, and 76.2% did not have AF. Mortality rates were highest in patients with IS and known AF; among these patients, 54.5% received oral anticoagulant (OAC) therapy in the 90 days pre‐stroke compared with 78.7% in the 90 days post‐stroke. Antiplatelet use among non‐AF stroke patients increased from 37.5% pre‐stroke to 87.3% post‐stroke; smaller increases in antiplatelet use were seen among other IS cases. Antihypertensives and statins were widely used pre‐ and post‐stroke among all IS cases. Our findings indicate that there is scope for improvement in the level of OAC prescribing to patients with known AF, which could potentially help reduce the future burden of stroke.

## 1 INTRODUCTION

Age‐specific incidence and mortality rates of stroke have been declining in all European countries, yet the number of people experiencing stroke is rising due to the ageing population and the strong age relationship with stroke risk.^1^ In the United Kingdom, stroke is the fourth largest cause of death, and more than 1 in 8 people admitted to hospital with stroke in England and Wales die within 30 days.^2^

Good management of cardiovascular and other stroke risk factors is needed to reduce the occurrence of stroke. Eighty‐five percent of strokes are ischaemic,^3^ and up to a third of these are caused by cardiac thromboembolism attributed to atrial fibrillation (AF).^4^, ^5^ Cardioembolic strokes are often severe, leading to substantial disability or death,^6^ yet up to two‐thirds could be preventable with use of oral anticoagulant (OAC) therapy in patients with AF.^7^ However, underuse of OAC therapy in patients with atrial fibrillation (AF) remains common.^8^, ^9^, ^10^, ^11^

Improving stroke prevention and outcomes is a key priority in the UK National Health Service (NHS) Long Term Plan.^12^ Understanding the pattern of medication use in patients at risk of stroke would help target efforts to improve stroke prevention. Using population‐based primary care data from 2016 to 2018, we aimed to describe pre‐ and post‐stroke cardiovascular medication use (with a focus on antithrombotic therapy), and 1‐year mortality in individuals hospitalised for IS in the United Kingdom: those with AF diagnosed pre‐stroke, those diagnosed with AF post‐stroke, and those without a diagnosis of AF.

## 2 METHODS

### 2.1 Data source

We used the IQVIA Medical Research Data‐UK (IMRD‐UK) primary care database, previously The Health Improvement Network. The database contains de‐identified longitudinal patient data entered by primary care practitioners (PCPs) during routine patient care. Clinical data are entered using Read codes, the standard clinical codes used in the NHS. Prescriptions issued by the PCP are recorded automatically, and information from secondary care is entered retrospectively. In the United Kingdom, chronic disease is managed in primary care, making the IMRD‐UK suitable for this study. Additionally, in another UK primary database similar to IMRD‐UK, cerebrovascular disease Read codes have a 93% positive predictive value (PPV).^13^ The IMRD‐UK covers around 6% of the UK population^14^ and is generalisable to the country as a whole.^15^ The study protocol was approved by the Independent Scientific Research Committee for IMRD‐UK (reference number 19THIN057). Data collection for IMRD‐UK was approved by the South East Multicentre Research Ethics Committee in 2003 and individual studies using IMRD‐UK data do not require separate ethical approval if only anonymised data are used.

### 2.2 Source population and identification of ischaemic stroke cases

The source population included individuals aged 20–89 years between 1 July 2016 and 30 June 2018, permanently registered with their PCP and with at least 3 years' registration after their first recorded prescription. We followed individuals from the date they met the study entry criteria (start of follow‐up), until the first coded IS entry, death, or the end of the observation period (September 2019), whichever came first. We included only hospitalised cases, identified as having a record of hospitalisation between 15 days pre‐stroke and 30 days post‐stroke. This was determined by manually reviewing patient records as described previously.^16^ The date of hospitalisation for the stroke was the index date, and we retained only cases aged ≥55 years. As members of the source population may have experienced a stroke before the start date, cases could have been either first or recurrent events.

### 2.3 Ischaemic stroke cases with/without AF

We divided IS cases into three groups: AF diagnosed pre‐stroke (‘AF pre‐stroke group’), patients newly diagnosed with AF either on the date of stroke or in the year after (‘AF post‐stroke group’), and patients without AF diagnosed pre‐stroke and without AF diagnosed in the year post‐stroke (‘non‐AF stroke group’). Atrial fibrillation was ascertained by the presence of an AF code. We excluded patients with a code for mitral stenosis or valve replacement surgery before the date of AF diagnosis or in the 2 weeks after.

### 2.4 Comorbidities and other patient variables

We obtained information on patient demographics (age and sex) and lifestyle factors (body mass index, smoking and alcohol intake) any time before the index date using the most recent recorded value/status as appropriate. Healthcare use was measured by the number of PCP visits, referrals and hospitalisations in the year before the index date. Information on comorbidities (including cardiovascular disease and risk factors, renal function and frailty) were determined any time before the index date; for a history of cerebrovascular disease, we looked at the time period before the start of follow‐up. Further details on the ascertainment of co‐variates have been published previously.^16^

### 2.5 Antithrombotics and other cardiovascular medications

We determined pre‐ and post‐stroke exposure to antithrombotics [OACs and antiplatelets (low‐dose aspirin and clopidogrel)] and other medications (including antihypertensives, diuretics, beta‐blockers, angiotensin‐converting enzyme inhibitors, angiotensin II receptor blockers, statins, digoxin, and antiarrhythmics) among stroke cases still alive 30 days post‐stroke, based on prescription records. Pre‐stroke medication exposure was defined as a prescription in the 90 days before the index date, and post‐stroke medication exposure as a prescription on the index date or in the 90 days after.

### 2.6 Mortality rates

To determine deaths following stroke, we followed IS cases from the index date until the date of death or 31 September 2019 (the last available data collection date), whichever came first.

### 2.7 Statistical analysis

The study was designed to be predominantly descriptive in nature with no formal statistical hypotheses. For each stroke group, pre‐ and post‐stroke cardiovascular medication use, as well as other patient characteristics, were described using frequency counts and percentages for categorical data and means with standard deviation (SD) for continuous data. We calculated mortality rates per 100 person‐years with 95% confidence intervals (CIs) by dividing the total number of deaths during follow‐up by the person‐years at risk. We compared mortality rates between the AF pre‐stroke group and non‐AF group, but not the AF post‐stroke group due to the potential of immortal time bias among these cases from the need to have survived long enough to have the post‐stroke work‐up for AF diagnosis. We performed stratified analyses by sex and age, and a sensitivity analysis where we re‐analysed medication use among the AF post‐stroke group changing the index date to the date of AF diagnosis. Analyses were undertaken using Stata version 12 (StataCorp. 2017).

## 3 RESULTS

### 3.1 Characteristics of the IS patients

We identified 3201 hospitalised stroke cases. The majority (76.2%, 2441/3201), were non‐AF stroke, 15.7% (*n* = 501) had AF pre‐stroke, and 8.1% (*n* = 259) were AF post‐stroke. In the latter group, 61.0% (*n* = 158) had AF diagnosed within 30 days of the stroke date, and 39.0% (*n* = 101) had AF diagnosed between 31 days and 12 months after the stroke date. Characteristics of stroke cases are shown in Table 1 (see also Table S1 for distribution of CHA_2_DS_2_‐VASc score). Males accounted for slightly more than half of each group. The non‐AF stroke group were youngest on average and had better renal function. The AF pre‐stroke group had the highest prevalence of moderate/severe frailty, cerebrovascular disease, cardiovascular disease and reduced renal function, while the AF post‐stroke group were more overweight/obese.

**TABLE 1:** Distribution of demographics, lifestyle factors, and healthcare use among hospitalised IS cases aged ≥55 years according to AF group

Hospitalised IS cases *N* = 3201	AF pre‐stroke *N* = 501	AF post‐stroke *N* = 259	Non‐AF stroke *N* = 2441
Males	292 (58.3)	137 (52.9)	1350 (55.3)
Mean age (SD), years)	78.1 (8.2)	76.4 (8.1)	72.6 (9.1)
Median age (IQR)	80 (73–85)	77 (71–83)	73 (65–80)
BMI (kg/m^2^)^a^			
15–19	29 (5.8)	5 (1.9)	101 (4.1)
20–24	132 (26.3)	56 (21.6)	620 (25.4)
25–29	184 (36.7)	97 (37.5)	931 (38.1)
≥30	141 (28.1)	93 (35.9)	715 (29.3)
Missing	15 (3.0)	8 (3.1)	74 (3.0)
Lifestyle factors^a^			
Smoking	47 (9.4)	35 (13.5)	513 (21.0)
Alcohol >20 units/week	24 (4.8)	21 (8.1)	197 (8.1)
Healthcare use^b^			
≥20 PCP visits	316 (63.1)	85 (32.8)	905 (37.1)
≥20 referrals	91 (18.2)	16 (6.2)	238 (9.8)
≥3 hospitalisations	84 (16.8)	21 (8.1)	266 (10.9)
History of cerebrovascular disease^c^			
Ischaemic stroke	100 (20.0)	24 (9.3)	315 (12.9)
Transient ischaemic attack	77 (15.4)	20 (7.7)	224 (9.2)
Haemorrhagic stroke (intracerebral and subarachnoid haemorrhage)/subdural haematoma	20 (4.0)	2 (0.8)	58 (2.4)
Comorbidities^d^			
Ischaemic heart disease	195 (38.9)	61 (23.6)	543 (22.2)
Myocardial infarction	91 (18.2)	25 (9.7)	247 (10.1)
Unstable angina	16 (3.2)	5 (1.9)	49 (2.0)
Revascularisation	68 (13.6	22 (8.5)	182 (7.5)
Deep vein thrombosis	58 (11.6)	31 (12.0)	253 (10.4)
Peripheral artery disease	48 (9.6)	12 (4.6)	183 (7.5)
Cancer	155 (30.9)	62 (23.9)	604 (24.7)
Pancreatic disease	11 (2.2)	3 (1.2)	48 (2.0)
Liver disease	33 (6.6)	12 (4.6)	159 (6.5)
Hypertension	358 (71.5)	184 (71.0)	1502 (61.5)
Hyperlipidaemia	172 (34.3)	82 (31.7)	797 (32.7)
Diabetes	135 (26.9)	55 (21.2)	612 (25.1)
Urogenital bleeding	93 (18.6)	25 (9.7)	309 (12.7)
Upper GI bleeding	78 (15.6)	21 (8.1)	285 (11.7)
Lower GI bleeding	68 (13.6)	19 (7.3)	238 (9.8)
Unspecified GI bleeding	28 (5.6)	3 (1.2)	45 (1.8)
Coagulopathies	21 (4.2)	6 (2.3)	48 (2.0)
CHA_2_DS_2_‐VASc score, mean (SD)	4.0 (1.7)	3.3 (1.5)	3.0 (1.7)
HAS‐BLED score, mean (SD)	1.6 (0.8)	1.3 (0.6)	1.3 (0.8)
Frailty			
Fit	39 (7.8)	85 (32.8)	744 (30.5)
Mild frailty	134 (26.7)	89 (34.4)	894 (36.6)
Moderate frailty	166 (33.1)	58 (22.4)	541 (22.2)
Severe frailty	162 (32.3)	27 (10.4)	262 (10.7)
Renal function (eGFR ml/min/1.73 m^2^)			
<15	4 (0.8)	2 (0.8)	7 (0.3)
15–29	13 (2.6)	7 (2.7)	45 (1.8)
30–59	157 (31.3)	59 (22.8)	499 (20.4)
60–89	259 (51.7)	143 (55.2)	1250 (51.2)
≥90	67 (13.4)	42 (16.2)	561 (23.0)
Missing	1 (0.2)	6 (2.3)	79 (3.2)

*Note*: Data are *n* (%) unless otherwise stated. The AF post‐stroke group were patients newly diagnosed with AF either on the date of stroke or in the year after.

Abbreviations: AF, atrial fibrillation; BMI, body mass index; eGFR, estimated glomerular filtration rate; GI, gastrointestinal bleeding; IS, ischaemic stroke; atrial fibrillation; NA, not applicable; PCP, primary care practitioner; SD, standard deviation.

^**a**^ Alcohol intake, BMI and smoking were determined any time before the index date using the most recent status/value as appropriate.

^b^ PCP visits, referrals and hospitalisations were determined in the year before the index date.

^c^ Before the start of follow‐up.

^d^ Comorbidities were determined any time before the index date.

### 3.2 Pre‐ and post‐stroke OAC use

**FIGURE 1:**
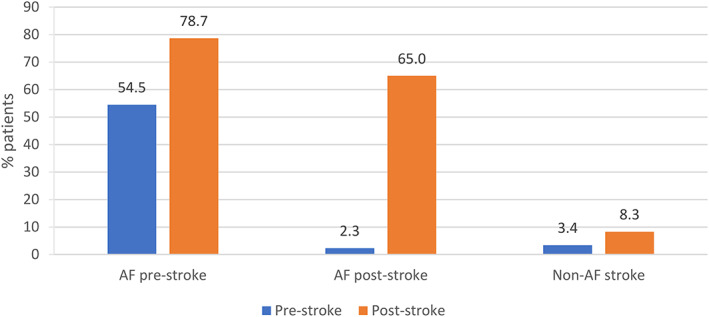
Pre‐ and post‐stroke OAC use among hospitalised IS cases aged ≥55 years according to AF group. AF, atrial fibrillation; IS, ischaemic stroke; OAC, oral anticoagulant.

In each group, OAC use increased in the post‐stroke period (Figure 1; Table 2). Just over half (54.3%) of the AF pre‐stroke group received OAC therapy pre‐stroke (this was broadly consistent among those with CHA_2_DS_2_‐VASc score 2–7; Table S2), rising to 78.7% post‐stroke. This increase was more evident in men (53.9%–81.4%) than in women (55.2%–74.9%), and in younger stroke cases (53.0%–83.4% for <75 years vs. 55.1% to 76.5% for ≥75 years; Table S3a). Among the AF pre‐stroke group, 133 of those prescribed an OAC in the 90 days post‐stroke were not OAC users in the 90 days pre‐stroke, and among these, the majority (*n* = 118) had either never used an OAC before their stroke or had discontinued more than a year before their stroke. Less than 4% of the other two stroke groups received an OAC pre‐stroke; however, 65.0% of the AF post‐stroke group received an OAC 90 days post‐stroke, and 84.8% received an OAC within 90 days of their AF diagnosis (93.4% of these being prescribed a NOAC; Table S3b). Among all stroke cases diagnosed with AF (AF pre‐stroke group and AF post‐stroke group), 80.8% received OAC therapy post‐AF diagnosis.

**TABLE 2:** OAC and other medication use in the 90 days pre‐stroke or in the 90 days post‐stroke among patients still alive 30 days after their stroke (*n* = 3119), according to AF group

	AF pre‐stroke *N* = 483		AF post‐stroke *N* = 257		Non‐AF stroke *N* = 2379	
	Pre‐stroke *n* (%)	Post‐stroke *n* (%)	Pre‐stroke *n* (%)	Post‐stroke *n* (%)	Pre‐stroke *n* (%)	Pre‐stroke *n* (%)
OAC	263 (54.5)	380 (78.7)	6 (2.3)	167 (65.0)	82 (3.4)	198 (8.3)
VKA	142 (29.4)	125 (25.9)	2 (0.8)	12 (4.7)	49 (2.1)	76 (3.2)
NOAC	129 (26.7)	313 (64.8)	5 (1.9)	156 (60.7)	34 (1.4)	134 (5.6)
Antiplatelet	149 (30.8)	171 (35.4)	99 (38.5)	141 (54.9)	892 (37.5)	2078 (87.3)
Low‐dose aspirin	10 (20.9)	100 (20.7)	61 (23.7)	62 (24.1)	601 (25.3)	724 (30.4)
Clopidogrel	54 (11.2)	94 (19.5)	43 (16.7)	104 (40.5)	363 (15.3)	1890 (79.4)
Antihypertensive	417 (86.3)	434 (89.9)	183 (71.2)	223 (86.8)	1485 (62.4)	1800 (75.7)
Diuretics	179 (37.1)	192 (39.8)	7 (30.4)	88 (34.2)	486 (20.4)	581 (24.4)
Beta‐blocker	288 (59.6)	311 (64.4)	87 (33.9)	13 (51.0)	548 (23.0)	612 (25.7)
ACE inhibitor	155 (32.1)	168 (34.8)	81 (31.5)	103 (40.1)	669 (28.1)	860 (36.1)
ARBs	75 (15.5)	76) (15.7)	31 (12.1)	34 (13.2)	315 (13.2)	348 (14.6)
Calcium‐channel blocker	137 (28.4)	166 (34.4)	86 (33.5)	107 (41.6)	673 (28.3)	936 (39.3)
Statin	269 (55.7)	378 (78.3)	136 (52.9)	216 (84.0)	1142 (48.0)	2062 (86.7)
Digoxin	92 (19.0)	103 (21.3)	0 (0.0)	10 (3.9)	8 (0.3)	1 (0.6)
Antiarrhythmic	36 (7.5)	31 (6.4)	10 (3.9)	13 (5.1)	65 (2.7)	70 (2.9)

*Note*: The AF post‐stroke group were patients newly diagnosed with AF either on the date of stroke or in the year after. The date of hospitalised IS was used as the index date for the AF post‐stroke group. *p* values are from Chi^2^ testing for differences in proportions between stroke categories.

Abbreviations: ACE, angiotensin‐converting enzyme; AF, atrial fibrillation; ARB, angiotensin II receptor blocker; IS, ischaemic stroke; NOAC, non‐vitamin K antagonist oral anticoagulant; OAC, oral anticoagulant; VKA, vitamin K antagonist.

### 3.3 Pre‐ and post‐stroke antiplatelet use

The proportion of stroke cases prescribed an antiplatelet pre‐stroke was broadly similar between groups with notable differences post‐stroke: 30.8% (pre‐stroke) to 35.4% (post‐stroke) in the AF pre‐stroke group, 38.5% (pre‐stroke) to 54.9% (post‐stroke) in the AF post‐stroke group (47.5% when using date of AF diagnosis as the index date), and 37.5% (pre‐stroke) to 87.3% (post‐stroke) in the non‐AF stroke group (Table 2; Figure S1a and Table S3a). Antiplatelet use according to whether this was prescribed with/without OAC therapy is shown in Table S4.

### 3.4 Pre‐ and post‐stroke use of other medications

Generally, use of all cardiovascular medications increased between the pre‐ and post‐stroke periods (Table 2), and was highest in the AF pre‐stroke group. Antihypertensives were commonly prescribed in each stroke group. Overall, 66.8% (2085/3119) of stroke cases received antihypertensives pre‐stroke rising to 78.8% (2457/3119) post‐stroke; increases were 86.3%–89.9% in the AF pre‐stroke group, 71.2–86.8% in the AF post‐stroke group, and 62.4%–75.7% in the non‐AF group (Figure S1b). Just under a third of patients in each group received ACE inhibitors pre‐stroke rising to over a third post‐stroke, and between 20.4% and 37.1% of patients in each group received diuretics pre‐stroke, increasing to between 24.4% and 39.8% post‐stroke. Statins were also commonly prescribed in each stroke group; 49.6% (1547/3119) stroke cases received statins pre‐stroke rising to 85.2% (2656/3119) post‐stroke; increases were 55.7%–78.3% in the AF pre‐stroke group, 52.9%–84.0% in the AF post‐stroke group, and 48.0%–86.7% in the non‐AF stroke group (Figure S1c).

### 3.5 Mortality

**FIGURE 2:**
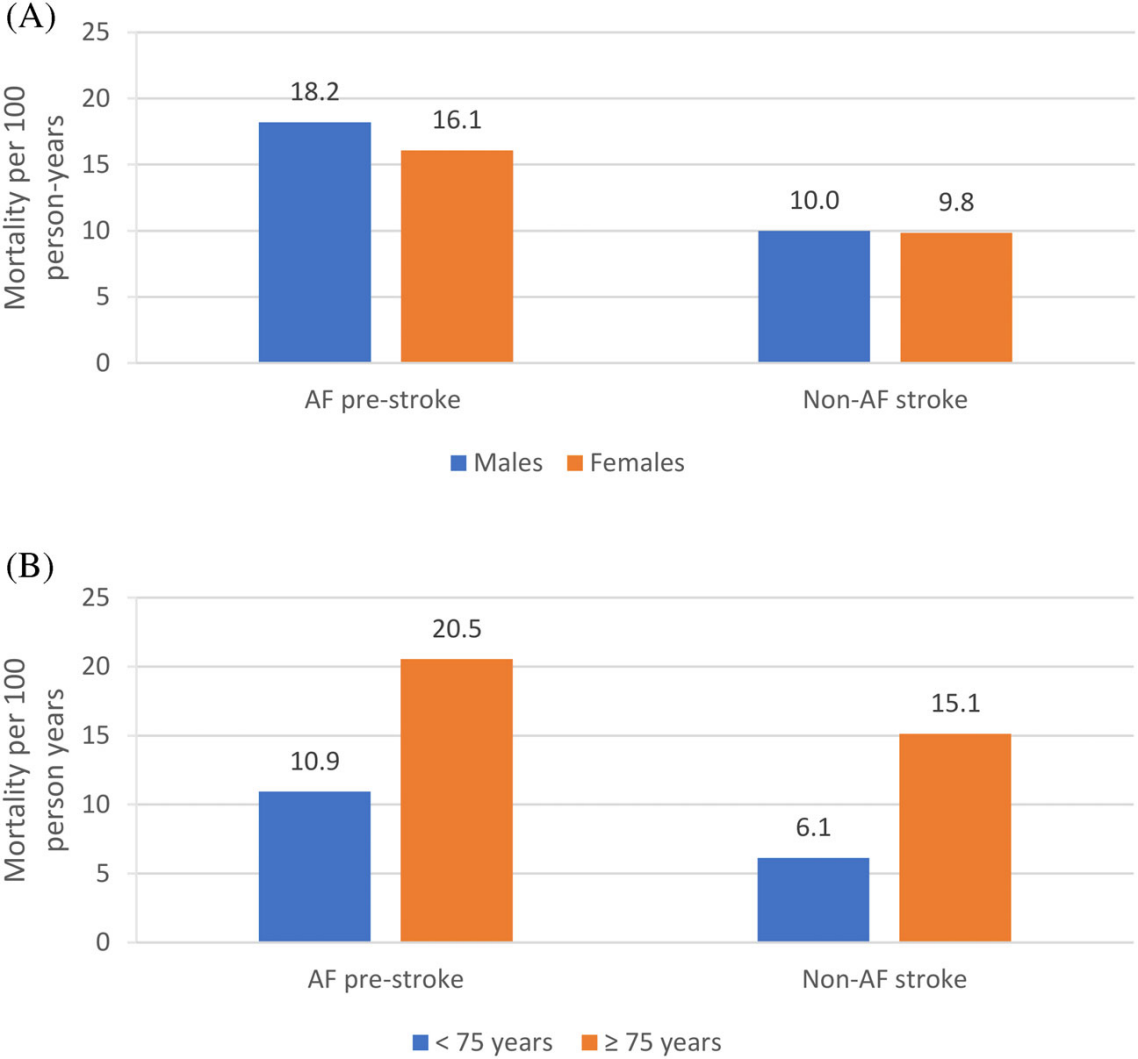
Mortality rate per 100 person‐years among the AF pre‐stroke group and the non‐AF stroke group, stratified by (A) sex and (B) age (<75 years, ≥75 years). AF, atrial fibrillation; IS, ischaemic stroke.

Six hundred and twelve people died during follow‐up. Mortality in the AF pre‐stroke group was almost double the rate among those in the non‐AF group: 17.30 per 100 person‐years (95% CI: 14.70–20.35) versus 9.91 (95% CI: 9.02–10.89). Mortality rates were slightly higher in males than females in the AF pre‐stroke group (18.2 vs. 16.1 per 100 person‐years) and were similar between the sexes in the non‐AF group (10.0 vs. 9.8 per 100 person‐years; Figure 2A). In the AF pre‐stroke group, the mortality rate among patients aged ≥75 years was almost double the rate in those aged <75 years (20.5 vs. 10.9 deaths per 100 person‐years), while in the non‐AF group it was almost triple (15.1 vs. 6.1 deaths per 100 person‐years; Figure 2B).

## 4 DISCUSSION

### 4.1 Summary

Among 3201 patients hospitalised with IS in this study, close to one in six had a pre‐stroke diagnosis of AF. These patients were, on average, older, frailer, and they more commonly received preventative cardiovascular medication pre‐stroke, and had a higher mortality rate than stroke cases without AF. Among the pre‐stroke AF group, 54.5% received OAC therapy in the 90 days pre‐stroke, compared with 78.7% in the 90 days post‐stroke. The majority (87.3%) of stroke cases without AF received post‐stroke antiplatelet therapy, which was a much higher level of use than among AF stroke cases, and, in general, use of other cardiovascular medications were seen to increase between the pre‐ and post‐stroke periods.

### 4.2 Strengths and limitations

Strengths of the study include the representative sample of the UK population, making the results generalisable to the country as a whole, the suitability of the database to capture long‐term use of cardiovascular medications, and the high 93% PPV of coded cerebrovascular entries.^13^ We did not evaluate whether OAC therapy was adequately dosed as this is challenging to establish for VKAs and was beyond the scope of the study. Our main analyses were not restricted to high‐risk patients, however, additional analysis by CHA_2_DS_2_VASc score in AF pre‐stroke patients showed pre‐stroke OAC use was broadly consistent between CHA_2_DS_2_VASc scores 2 and 9 (covering 92% of those prescribed OACs). As the vast majority of the AF pre‐stroke group had a CHA_2_DS_2_VASc score ≥2, perceived low risk of stroke is unlikely to account for much of the observed pre‐stroke underuse of OACs in patients with known AF; possible explanatory reasons for OAC underuse—either pre‐ or post‐stroke—might include patient preference, contraindications, and terminal illness. Prescriptions issued in hospital will not have been captured, but our 90‐day window to determine long‐term post‐stroke medication use means that exposure misclassification is likely minimal. Although previous reports have found OAC discontinuation to be common,^17^, ^18^ only 11% of the AF pre‐stroke group (*n* = 25) not prescribed an OAC pre‐stroke were recent OAC discontinuers. Communications gaps between primary and secondary care may have led to some misclassification of AF status, for example, AF diagnosed during the hospital episode not later recorded in the primary care record.

### 4.3 Comparison with existing literature

In the most recent report from the Sentinel Stroke National Audit Program (SSNAP), which covers around 90% of stroke patient hospital admissions in England, Wales and Northern Ireland, 54%–64% of patients with AF hospitalised for stroke between August 2016 and March 2019 received OAC therapy pre‐admission, which is consistent with our estimate.^19^ This level of pre‐stroke OAC use in patients with AF is also broadly in line with findings from Denmark (41.5%–58.5%)^20^, ^21^ and Sweden (41.2%).^22^ It is also notably higher than estimates from the United Kingdom over earlier time periods in the United Kingdom^11^, ^19^; for example, in the SSNAP, 40% of hospitalised stroke patients with AF received OAC therapy during April to June 2014.^19^ Our data therefore signify an improvement in OAC prescribing in AF, in line with the implementation of National Quality Improvement initiatives to increase uptake, yet still with scope for improvement. The SSNAP reported that 98% of patients discharged from hospital for stroke and with AF were prescribed anticoagulation to reduce the risk of recurrent stroke, which is substantially higher than the 80.8% of patients in our study (where AF was diagnosed either pre‐ or post‐stroke). This suggests that efforts should be made to ensure patients with AF continue with OAC therapy post hospital discharge for stroke. However, we found that OAC use following a new diagnosis of AF post‐stroke was high (84.8% within 90 days after AF diagnosis).

Antihypertensives and statins were widely prescribed post‐stroke (79% and 85%, respectively) in line with national guidelines. They were also quite widely prescribed pre‐stroke (67% and 50%, respectively). Pre‐stroke statin use could, however, be considered lower than some might expect considering that the median age of stroke cases was 71–80 years, and that UK guidance advocates statins for both primary and secondary stroke prevention in patients with diabetes or a ≥10% risk of developing CVD within 10 years.^23^ Comparisons with other literature on this topic are limited. Using UK primary care data for 2008, Lee et al^11^ found that pre‐stroke use of antihypertensives and lipid‐regulating drugs was ~64% and ~30%, respectively, while post‐stroke use was around ~72% and ~80%, respectively.

## CONFLICT OF INTEREST

LAGR works for CEIFE, which has received research funding Bayer AG. LAGR has also received honoraria for serving on advisory boards for Bayer AG. David Gaist has received honoraria from AstraZeneca (Sweden) for participation as a coinvestigator on a research project outside the submitted work; and receiving speaker honorarium from Bristol‐Myers Squibb outside the submitted work. Yanina Balabanova is an employee of Bayer AG. Gunnar Brobert is a former employee of Bayer AB and is currently a paid consultant for Bayer. Mike Sharma has served on the steering committees and led sub‐studies from trials sponsored by Bayer and has served as a consultant and received speaker's honoraria from Bayer. Mike Sharma has also served as a consultant to Portola, Bristol Myers Squibb and Janssen. Lucia Cea Soriano has no potential competing interests.

## ETHICS STATEMENT

The study protocol was approved by the Independent Scientific Research Committee for IMRD‐UK (reference number 19THIN057). Data collection for IMRD‐UK was approved by the South East Multicentre Research Ethics Committee in 2003 and individual studies using IMRD‐UK data do not require separate ethical approval if only anonymised data are used.

## Supporting information


**Appendix S1** Supporting Information.
