# Supplementary material for: Pre‐ and post‐stroke oral antithrombotics and mortality in patients with ischaemic stroke
Source: Pharmacoepidemiol Drug Saf. 2022 Sep 9;31(11):1182–9. doi: 10.1002/pds.5530 (PMC9825966; doi:10.1002/pds.5530)
Supplement: Supplementary file 1 — Appendix S1 Supporting Information. [file PDS-31-1182-s001.pdf]

Supplementary Table 1. Distribution of CHA_2DS_2-VASc score in each stroke group.

**Hospitalized IS cases N=3201**	**AF pre-stroke N=501**	**AF post-stroke N=259**	**Non-AF stroke N=2441**
**CHA_2DS_2-VASc score**			
Mean (SD)	4.0 (1.7)	3.3. (1.5)	3.0 (1.7)
0	14 (2.8)	14 (5.4)	197 (8.1)
1	20 (4.0)	15 (5.8)	239 (9.8)
2	61 (12.2)	39 (15.1)	494 (20.2)
3	95 (19.0)	80 (30.9)	566 (23.2)
4	132 (26.3)	57 (22.0)	530 (21.7)
5	92 (18.4)	34 (13.1)	247 (10.1)
6	53 (10.6)	17 (6.6)	122 (5.0)
7	20 (4.0)	3 (1.2)	25 (1.0)
8	9 (1.8)	0 (0.0)	19 (0.8)
9	5 (1.0)	0 (0.0)	2 (0.1)
≥4	311 (62.1)	111 (42.9)	945 (38.7)

Data are n (%) unless otherwise stated.

*As a continuous variable.

Supplementary Table 2. OAC use in the 90 days pre-stroke among AF pre-stroke cases still alive 30 days after their stroke.

**OAC use pre-stroke**	**AF pre-stroke N=483**	
**All cases, n**	**483**	
OAC use, %	263	54.5
**CHA_2DS_2-VASc 0, n**	**14**	
OAC use, %	3	21.4
**CHA_2DS_2-VASc 1, n**	**20**	
OAC, %	8	40.0
**CHA_2DS_2-VASc 2, n**	**60**	
OAC use, %	32	53.3
**CHA_2DS_2-VASc 3, n**	**92**	
OAC, %	52	56.5
**CHA_2DS_2-VASc 4, n**	**128**	
OAC use, %	67	52.3
**CHA_2DS_2-VASc 5 =, n**	**88**	
OAC use, %	52	59.1
**CHA_2DS_2-VASc 6, n**	**50**	
OAC use, %	29	58.0
**CHA_2DS_2-VASc 7, n**	**19**	
OAC use, %	10	52.6
**CHA_2DS_2-VASc 8, n**	**7**	
OAC use, %	5	71.4
**CHA_2DS_2-VASc 9, n**	**5**	
OAC use, %	5	100.0

Supplementary Table 3a. Antithrombotic and other cardiovascular medication use in the 90 days pre-stroke or in the 90 days post-stroke among hospitalised IS cases still alive 30 days after their stroke, according to AF group, and stratified by sex and age.
Note: using the date of hospitalised IS as the index date for the AF post-stroke group.

**Medication**	**AF pre-stroke N=483**				**AF post-stroke N=257**				**Non-AF stroke N=2379**			
	**Pre-stroke**		**Post-stroke**		**Pre-stroke**		**Post-stroke**		**Pre-stroke**		**Post-stroke**	
	**n**	**%**	**n**	**%**	**n**	**%**	**n**	**%**	**n**	**%**	**n**	**%**
**OACs**	263	54.5	380	78.7	6	2.3	167	65.0	82	3.4	198	8.3
Men	151	53.9	228	81.4	5	3.6	92	67.2	42	3.2	104	7.9
Women	112	55.2	152	74.9	1	0.8	75	62.5	40	3.8	94	8.9
<75 years	80	53.0	126	83.4	0	0.0	72	67.9	38	2.9	88	6.6
≥75 years	183	55.1	254	76.5	6	4.0	95	62.9	44	4.2	110	10.5
**VKAs**	142	29.4	125	25.9	2	0.8	12	4.7	49	2.1	76	3.2
Men	84	30.0	81	28.9	2	1.5	8	5.8	32	2.4	48	3.6
Women	58	28.6	44	21.7	0	0.0	4	3.3	17	1.6	28	2.6
<75 years	41	27.2	37	24.5	0	0.0	4	3.8	24	1.8	40	3.0
≥75 years	101	30.4	88	26.5	2	1.3	8	5.3	25	2.4	36	3.4
**NOACs**	129	26.7	313	64.8	5	1.9	156	60.7	34	1.4	134	5.6
Men	74	26.4	189	67.5	4	2.9	85	62.0	11	0.8	63	4.8
Women	55	27.1	124	61.1	1	0.8	71	59.2	23	2.2	71	6.7
<75 years	41	27.2	106	70.2	0	0.0	68	64.2	14	1.1	56	4.2
≥75 years	88	26.5	207	62.3	5	3.3	118	78.1	20	1.9	78	7.4
**Antiplatelets**	149	30.8	171	35.4	99	38.5	141	54.9	892	37.5	2078	87.3
Men	94	33.6	101	36.1	55	40.1	79	57.7	512	38.9	1157	87.9
Women	55	27.1	70	34.5	44	36.7	62	51.7	380	35.8	921	86.7
<75 years	38	25.2	48	31.8	35	33.0	49	46.2	437	32.9	1180	88.9
≥75 years	111	33.4	123	37.0	64	42.4	79	52.3	455	43.3	898	85.4
**Low-dose aspirin**	101	20.9	100	20.7	61	23.7	62	24.1	601	25.3	724	30.4
Men	67	23.9	63	22.5	33	24.1	34	24.8	356	27.0	430	32.6
Women	34	16.7	37	18.2	28	23.3	28	23.3	245	23.1	294	27.7
<75 years	25	16.6	26	17.2	21	19.8	22	20.8	275	20.7	375	28.3

Medication	AF pre-stroke N=483				AF post-stroke N=257				Non-AF stroke N=2379			
	Pre-stroke		Post-stroke		Pre-stroke		Post-stroke		Pre-stroke		Post-stroke	
	n	%	n	%	n	%	n	%	n	%	n	%
≥75 years	76	22.9	74	22.3	40	26.5	40	26.5	326	31.0	349	33.2
**Clopidogrel**	54	11.2	94	19.5	43	16.7	104	40.5	363	15.3	1890	79.4
Men	30	10.7	53	18.9	24	17.5	57	41.6	204	15.5	1062	80.6
Women	24	11.8	41	20.2	19	15.8	47	39.2	159	15.0	828	78.0
<75 years	14	9.3	30	19.9	15	14.2	40	37.7	202	15.2	1100	82.9
≥75 years	40	12.0	64	19.3	28	18.5	64	42.4	161	15.3	790	75.1
**Antihypertensives**	417	86.3	434	89.9	183	71.2	223	86.8	1485	62.4	1800	75.7
Men	240	85.7	255	91.1	101	73.7	124	90.5	836	63.5	1015	77.1
Women	177	87.2	179	88.2	82	68.3	99	82.5	649	61.1	785	73.9
<75 years	130	86.1	138	91.4	72	67.9	96	90.6	761	57.3	994	74.9
≥75 years	287	86.4	296	89.2	111	73.5	127	84.1	724	68.8	806	76.6
**Diuretics**	179	37.1	192	39.8	78	30.4	88	34.2	486	20.4	581	24.4
Men	90	32.1	104	37.1	36	26.3	44	32.1	228	17.3	278	21.1
Women	89	43.8	88	43.3	42	35.0	44	36.7	258	24.3	303	28.5
<75 years	43	28.5	51	33.8	26	24.5	33	31.1	203	15.3	269	20.3
≥75 years	136	41.0	141	42.5	52	34.4	54	35.8	283	26.9	312	29.7
**Beta-blockers**	288	59.6	311	64.4	87	33.9	131	51.0	548	23.0	612	25.7
Men	160	57.1	174	62.1	43	31.4	63	46.0	283	21.5	322	24.4
Women	128	63.1	137	67.5	44	36.7	68	56.7	265	25.0	290	27.3
<75 years	95	62.9	106	70.2	37	34.9	58	54.7	269	20.3	314	23.7
≥75 years	193	58.1	205	61.7	50	33.1	73	48.3	279	26.5	298	28.3
**ACE inhibitors**	155	32.1	168	34.8	81	31.5	103	40.1	669	28.1	860	36.1
Men	97	34.6	104	37.1	47	34.3	61	44.5	410	31.1	532	40.4
Women	58	28.6	64	31.5	34	28.3	42	35.0	259	24.4	328	30.9
<75 years	50	33.1	57	37.7	36	34.0	52	49.1	376	28.3	521	39.3
≥75 years	105	31.6	111	33.4	45	29.8	51	33.8	293	27.9	339	32.2
**ARBs**	75	15.5	76	15.7	31	12.1	34	13.2	315	13.2	348	14.6

Medication	AF pre-stroke N=483				AF post-stroke N=257				Non-AF stroke N=2379			
	Pre-stroke		Post-stroke		Pre-stroke		Post-stroke		Pre-stroke		Post-stroke	
	n	%	n	%	n	%	n	%	n	%	n	%
Men	46	16.4	48	17.1	19	13.9	22	16.1	161	12.2	180	13.7
Women	29	14.3	28	13.8	12	10.0	12	10.0	154	14.5	168	15.8
<75 years	23	15.2	25	16.6	10	9.4	13	12.3	160	12.1	192	14.5
≥75 years	52	15.7	51	15.4	21	13.9	21	13.9	155	14.7	156	14.8
**Calcium-channel blockers**	137	28.4	166	34.4	86	33.5	107	41.6	673	28.3	936	39.3
Men	83	29.6	102	36.4	53	38.7	63	46.0	392	29.8	537	40.8
Women	54	26.6	64	31.5	33	27.5	44	36.7	281	26.5	399	37.6
<75 years	43	28.5	52	34.4	36	34.0	44	41.5	366	27.6	534	40.2
≥75 years	94	28.3	114	34.3	50	33.1	63	41.7	307	29.2	402	38.2
**Statins**	269	55.7	378	78.3	136	52.9	216	84.0	1142	48.0	2062	86.7
Men	168	60.0	231	82.5	78	56.9	122	89.1	676	51.3	1166	88.5
Women	101	49.8	147	72.4	58	48.3	94	78.3	466	43.9	896	84.4
<75 years	93	61.6	136	90.1	57	53.8	96	90.6	626	47.2	1186	89.4
≥75 years	176	53.0	242	72.9	79	52.3	120	79.5	516	49.0	876	83.3
**Digoxin**	92	19.0	103	21.3	0	0.0	10	3.9	8	0.3	14	0.6
Men	49	17.5	54	19.3	0	0.0	2	1.5	5	0.4	7	0.5
Women	43	21.2	49	24.1	0	0.0	8	6.7	3	0.3	7	0.7
<75 years	28	18.5	31	20.5	0	0.0	5	4.7	4	0.3	5	0.4
≥75 years	64	19.3	72	21.7	0	0.0	5	3.3	4	0.4	9	0.9
**Antiarrhythmics**	36	7.5	31	6.4	10	3.9	13	5.1	65	2.7	70	2.9
Men	18	18	16	5.7	6	4.4	8	5.8	38	2.9	41	3.1
Women	18	8.9	15	7.4	4	3.3	5	4.2	27	2.5	29	2.7
<75 years	9	6.0	9	6.0	8	7.5	9	8.5	33	2.5	38	2.9
≥75 years	27	8.1	22	6.6	2	1.3	4	2.7	32	3.0	32	3.0

ACE, angiotensin-converting enzyme; AF, atrial fibrillation; ARB, angiotensin II receptor blocker; IS, ischaemic stroke; NA, not applicable; NOAC, non-vitamin K antagonist oral anticoagulant; OAC, oral anticoagulant; VKA, vitamin K antagonist

Supplementary Table 3b (sensitivity analysis). Antithrombotic and other cardiovascular medication use in the 90 days pre-stroke or in the 90 days post-stroke among hospitalised IS cases still alive 30 days after their stroke, among the AF post-stroke group.
Note: using the date of hospitalised stroke as the index date and the date of AF as the index date for the AF post-stroke group.

	**AF post-stroke (index date=date of IS) N=257**				**AF post-stroke (index date=date of AF) N=257**			
**Medication use**	**Pre-stroke**		**Post-stroke**		**Pre-stroke**		**Post-stroke**	
	**n**	**%**	**n**	**%**	**n**	**%**	**n**	**%**
**OACs**	6	2.3	167	65.0	23	8.9	218	84.8
Men	5	3.6	92	67.2	15	10.8	122	89.1
Women	1	0.8	75	62.5	8	6.7	96	80.0
<75 years	0	0.0	72	67.9	8	7.4	93	87.7
≥75 years	6	4.0	95	62.9	15	9.9	125	82.8
**VKAs**	2	0.8	12	4.7	2	0.8	14	5.4
Men	2	1.5	8	5.8	1	0.7	8	5.8
Women	0	0.0	4	3.3	1	0.8	6	5.0
<75 years	0	0.0	4	3.8	2	1.9	7	6.6
≥75 years	2	1.3	8	5.3	0	0.0	7	4.6
**NOACS**	5	1.9	156	60.7	21	8.2	205	79.8
Men	4	2.9	85	62.0	14	10.2	115	83.9
Women	1	0.8	71	59.2	7	5.8	90	75.0
<75 years	0	0.0	68	64.2	6	5.7	87	82.1
≥75 years	5	3.3	118	78.1	15	9.9	118	78.1
**Antiplatelets**	99	38.5	141	54.9	136	52.9	122	47.5
Men	55	40.1	79	57.7	78	56.9	65	47.4
Women	44	36.7	62	51.7	58	48.3	57	47.5
<75 years	35	33.0	49	46.2	46	43.4	43	40.6
≥75 years	64	42.4	79	52.3	90	59.6	79	52.3
**Low-dose aspirin**	61	23.7	62	24.1	54	21.0	40	15.6
Men	33	24.1	34	24.8	28	20.4	19	13.9
Women	28	23.3	28	23.3	26	21.7	21	17.5
<75 years	21	19.8	22	20.8	18	17.0	9	8.5
≥75 years	40	26.5	40	26.5	36	23.8	31	20.5
**Clopidogrel**	43	16.7	104	40.5	97	37.7	92	35.8
Men	24	17.5	57	41.6	55	40.1	49	35.8
Women	19	15.8	47	39.2	42	35.0	43	35.8
<75 years	15	14.2	40	37.7	35	33.0	37	34.9
≥75 years	28	18.5	64	42.4	62	41.1	55	36.4
**Antihypertensives**	183	71.2	223	86.8	192	74.7	223	86.8
Men	101	73.7	124	90.5	106	77.4	125	91.2
Women	82	68.3	99	82.5	86	71.7	98	81.7
<75 years	72	67.9	96	90.6	76	71.7	97	91.5
≥75 years	111	73.5	127	84.1	116	76.8	126	83.4
**Diuretics**	78	30.4	88	34.2	82	31.9	87	33.9
Men	36	26.3	44	32.1	39	28.5	45	32.8
Women	42	35.0	44	36.7	43	35.8	42	35.0
<75 years	26	24.5	33	31.1	30	28.3	33	31.1
≥75 years	52	34.4	54	35.8	52	34.4	54	35.8
**Beta-blockers**	87	33.9	131	51.0	91	35.4	141	54.9

	AF post-stroke (index date=date of IS) N=257				AF post-stroke (index date=date of AF) N=257			
**Medication use**	**Pre-stroke**		**Post-stroke**		**Pre-stroke**		**Post-stroke**	
	**n**	**%**	**n**	**%**	**n**	**%**	**n**	**%**
Men	43	31.4	63	46.0	45	32.8	71	51.8
Women	44	36.7	68	56.7	46	38.3	70	58.3
<75 years	37	34.9	58	54.7	40	37.7	64	60.4
≥75 years	50	33.1	73	48.3	51	33.8	77	51.0
**ACE inhibitors**	81	31.5	103	40.1	85	33.1	94	36.6
Men	47	34.3	61	44.5	48	35.0	54	39.4
Women	34	28.3	42	35.0	37	30.8	40	33.3
<75 years	36	34.0	52	49.1	40	37.7	50	47.2
≥75 years	45	29.8	51	33.8	45	29.8	44	29.1
**ARBs**	31	12.1	34	13.2	32	12.4	35	13.6
Men	19	13.9	22	16.1	19	13.9	22	16.1
Women	12	10.0	12	10.0	12	10.0	13	10.8
<75 years	10	9.4	13	12.3	10	9.4	14	13.2
≥75 years	21	13.9	21	13.9	21	13.9	21	13.9
**Calcium-channel blockers**	86	33.5	107	41.6	95	37.0	104	40.5
Men	53	38.7	63	46.0	54	39.4	61	44.5
Women	33	27.5	44	36.7	41	34.2	43	35.8
<75 years	36	34.0	44	41.5	37	34.9	43	40.6
≥75 years	50	33.1	63	41.7	58	38.4	61	40.4
**Statins**	136	52.9	216	84.0	167	64.5	212	82.5
Men	78	56.9	122	89.1	95	69.3	121	88.3
Women	58	48.3	94	78.3	71	59.2	91	75.8
<75 years	57	53.8	96	90.6	68	64.2	96	90.6
≥75 years	79	52.3	120	79.5	98	64.9	116	76.8
**Digoxin**	0	0.0	10	3.9	0	0.0	14	5.4
Men	0	0.0	2	1.5	0	0.0	3	2.2
Women	0	0.0	8	6.7	0	0.0	11	9.2
<75 years	0	0.0	5	4.7	0	0.0	7	6.6
≥75 years	0	0.0	5	3.3	0	0.0	7	4.6
**Antiarrhythmics**	10	3.9	13	5.1	10	3.9	13	5.1
Men	6	4.4	8	5.8	6	4.4	8	5.8
Women	4	3.3	5	4.2	4	3.3	5	4.2
<75 years	8	7.5	9	8.5	8	7.5	9	8.5
≥75 years	2	1.3	4	2.7	2	1.3	4	2.7

ACE, angiotensin-converting enzyme; AF, atrial fibrillation; ARB, angiotensin II receptor blocker; IS, ischaemic stroke; NOAC, non-vitamin K antagonist oral anticoagulant; OAC, oral anticoagulant; VKA, vitamin K antagonist

Supplementary Table 4. OAC and antiplatelet use in the 90 days pre-stroke or in the 90 days post-stroke among hospitalised IS cases still alive 30 days after their stroke, according to AF group.

**OAC use**	**AF pre-stroke N=483**				**AF post-stroke N=257**				**Non-AF stroke N=2379**			
	**Pre-stroke**		**Post-stroke**		**Pre-stroke**		**Post-stroke**		**Pre-stroke**		**Post-stroke**	
	**n**	**%**	**n**	**%**	**n**	**%**	**n**	**%**	**n**	**%**	**n**	**%**
No OAC, no antiplatelet	102	21.1	29	6.0	154	59.9	18	7.0	1417	59.6	180	7.6
OAC+antiplatelet	31	6.4	97	20.1	2	0.8	69	26.9	12	0.5	77	3.2
Antiplatelet only	118	24.4	74	15.3	97	37.7	72	28.0	880	37.0	2001	84.1
OAC only	232	48.0	283	58.6	4	1.6	98	38.1	70	2.9	121	5.1

AF, atrial fibrillation; IS, ischaemic stroke; OAC, oral anticoagulant

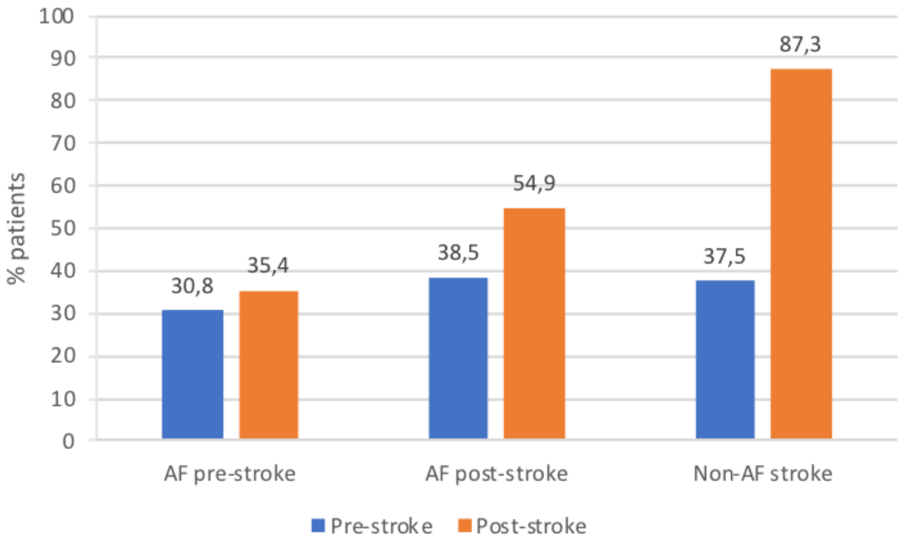

Supplementary Figure 1a. Pre- and post-stroke antiplatelet use among hospitalised IS cases aged ≥55 years according to AF group.
AF, atrial fibrillation; IS, ischaemic stroke

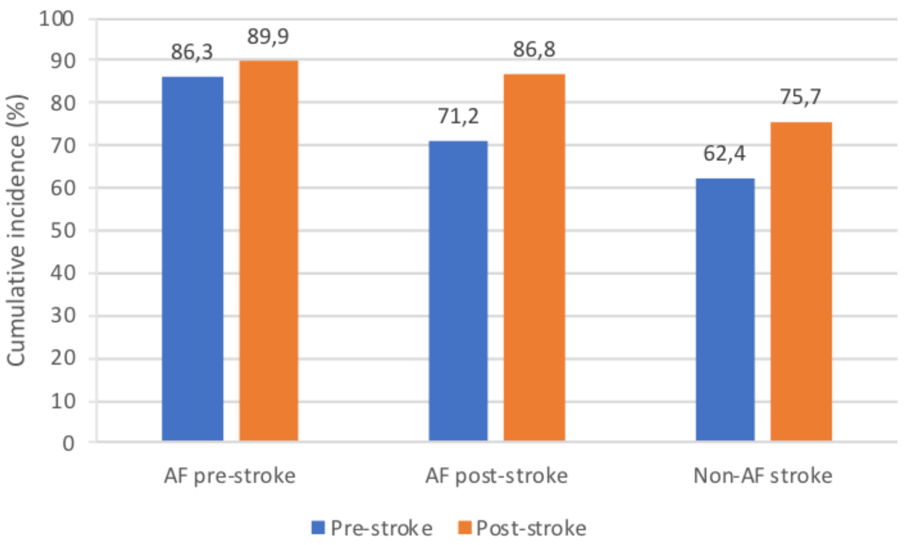

Supplementary Figure 1b. Pre- and post-stroke antihypertensive use among hospitalised IS cases aged ≥55 years according to AF group.
AF, atrial fibrillation; IS, ischaemic stroke

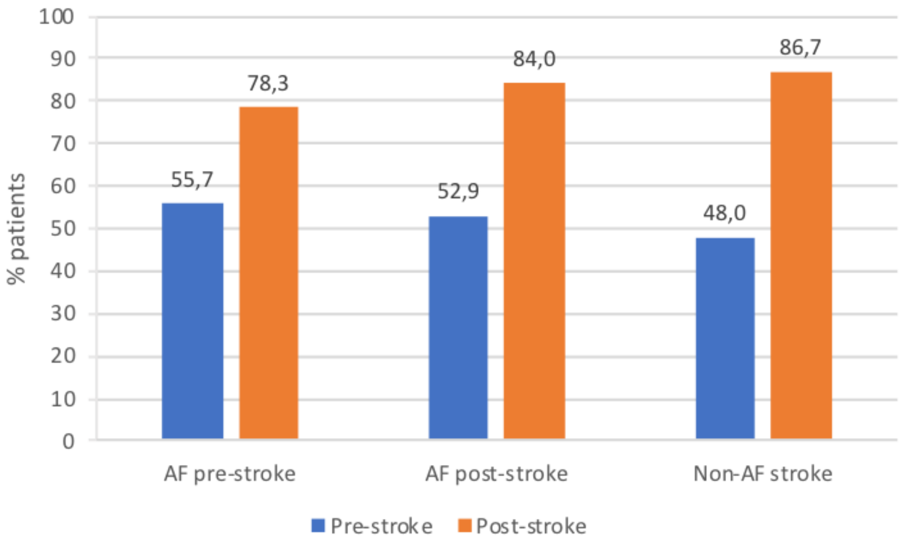

Supplementary Figure 1c. Pre- and post-stroke statin use among hospitalised IS cases aged ≥55 years according to AF group.
AF, atrial fibrillation; IS, ischaemic stroke
